# Evaluation of Abdominal Musculature Thickness, Pelvic Tilt, and Trunk Mobility in Women with Primary Dysmenorrhea: A Cross-Sectional Observational Study

**DOI:** 10.3390/jcm13133817

**Published:** 2024-06-29

**Authors:** Rebeca del Prado-Álvarez, Cecilia Estrada-Barranco, Ángel González-de-la-Flor, María-José Giménez, Marta de la Plaza San Frutos, Jaime Almazán-Polo, María García-Arrabé

**Affiliations:** Department of Physiotherapy, Faculty of Sport Sciences, Universidad Europea de Madrid, Villaviciosa de Odón, 28670 Madrid, Spain; rebeca.delprado@universidadeuropea.es (R.d.P.-Á.); cecilia.estrada@universidadeuropea.es (C.E.-B.); angel.gonzalez@universidadeuropea.es (Á.G.-d.-l.-F.); marta.delaplaza@universidadeuropea.es (M.d.l.P.S.F.); jaime.almazan@universidadeuropea.es (J.A.-P.); maria.gararrabe@universidadeuropea.es (M.G.-A.)

**Keywords:** dysmenorrhea, core, abdominal muscle, mobility, pelvic tilt, ultrasound

## Abstract

**Background**: This cross-sectional observational study aimed to investigate differences in abdominal musculature thickness, pelvic tilt, and trunk mobility between women with primary dysmenorrhea (PD) and a control group (CG). **Methods**: Participants included 44 women (22 with PD and 22 controls) aged over 18, nulliparous, and of reproductive age. Ultrasound imaging was used to measure the thickness of the transverse abdominis (TrA), internal oblique (IO), external oblique (EO), and rectus abdominis (RA) muscles at rest and during contraction. Additionally, anterior pelvic tilt was assessed using the Palpation Meter (PALM), and trunk flexion and extension were measured using an accelerometer (activForce2). **Results**: Significant differences (*p* < 0.05) were found in RA and EO muscle thickness, with lower values in the PD group compared to CG. However, there were no significant differences (*p* > 0.05) in TrA and IO muscle thickness, anterior pelvic tilt, or trunk mobility between groups. **Conclusions**: These findings contribute to understanding the musculoskeletal factors potentially involved in dysmenorrhea. Further research is needed to explore associations between PD and structural and alignment parameters.

## 1. Introduction

Primary dysmenorrhea (PD), a common disorder among women of childbearing age, affects a substantial proportion of this population, with incidence rates ranging from 25% to 90% during ovulatory cycles [1]. This condition typically manifests within the first 48 to 72 h of menstruation, with approximately 10% of affected women experiencing debilitating symptoms [2]. PD is characterized by menstrual pain that occurs in the absence of any identifiable organic cause [3]. This phenomenon significantly affects patients’ quality of life, with recurrent symptoms such as abdominopelvic and lower back pain. Despite advances in pharmacological treatment, gaps remain in the understanding of the underlying mechanisms of PD, particularly in relation to the musculoskeletal system.

Exploring theories on the etiology of PD is essential to understand their underlying mechanisms. These theories involve hormonal, inflammatory, and neurotransmitter-related aspects. For example, hormonal fluctuations, particularly those related to prostaglandins, have been shown to play an important role in the pathogenesis of dysmenorrhea [4]. Elevated levels of inflammatory mediators are also associated with increased menstrual pain [4].

Furthermore, it has been suggested that spinal alignment and muscular disturbances in the pelvic region may be involved in the etiology of PD. Holtzman et al. [5] hypothesized that PD may be caused by spinal misalignment and reported possible beneficial effects of applying manual therapy to the sacral region, decreasing symptomatology in women with PD. Latthe et al. [6] concluded that regular exercise would decrease the incidence of dysmenorrhea; however, the results of the retrospective study by Blakey et al. [3] with 597 students were inconclusive. Thus, there is a lack of consensus in the findings on the relationship between PD and the structural and functional components of the musculoskeletal system.

Anterior pelvic tilt refers to a pelvic position in which the front of the pelvis tilts forward and downward while the back of the pelvis is elevated [7]. This can result in excessive hyperlordosis in the lumbar region of the spine and a pelvis position that tilts forward in relation to the hips [8]. Anterior pelvic tilt may be associated with muscle imbalances, weakness in the abdominal muscles, and excessive tension in the lumbar spine musculature, which may contribute to postural problems and discomfort in this region [9]. 

In the study by Dogan et al. [10], the primary objective was to reduce pain, muscle spasms, and improve kinesthetic awareness. To this end, the use of kinesiotape was suggested as a therapeutic option. However, it remains uncertain whether there is any mobility limitation in the specific region in women with dysmenorrhea.

Although several studies have addressed the assessment of the abdominal wall’s architecture using ultrasound to measure size, thickness, or even cross-sectional area (CSA) [11,12], few studies have focused on assessment during muscle contraction. The straight leg raise (SLR) test induces contraction of the abdominal musculature to stabilize the trunk [13], which highlights the importance of assessing the musculature not only at rest but also during contraction. This approach is crucial to understanding whether dysmenorrhea affects not only the muscle structure but also its ability to contract muscle fibers.

Given the potential implications of musculoskeletal factors in PD, further studies on pelvic posture, trunk mobility, and the structure of the abdominal muscular system are needed in women with PD. It is suggested that pain experienced by women with PD may be associated with changes in the structure, function, and mobility of the abdomino-lumbo-pelvic region. 

The aim of the present study was to assess pelvic anteversion, sagittal plane mobility (trunk flexion-extension), and thickness of the abdominal muscles: transversus abdominis (TrA), internal oblique (IO), external oblique (EO), and rectus abdominis (RA) in women with PD compared to those without dysmenorrhea, which could provide information about more effective and personalized therapeutic approaches for this clinical condition.

## 2. Methods

### 2.1. Research Design

A cross-sectional, observational study, with the analysts blinded to the data, was carried out at the European University of Madrid (UEM) in Madrid, Spain, from September 2022 to March 2023. The study adhered to the STrengthening the Reporting of OBservational studies in Epidemiology (STROBE) guidelines for reporting [13].

The local Research and Ethics Committee of the UEM approved the study protocol with code CIPI/23.146. The study was conducted in accordance with the principles described in the Declaration of Helsinki, and all participants provided written informed consent.

### 2.2. Subjects

The inclusion criteria required that participants be women with a diagnosis of PD confirmed by a doctor from the UEM medical service, over the age of 18, of reproductive age, and nulliparous. Exclusion criteria included the use of hormonal contraceptives, a diagnosis of endometriosis or any other urogynecological pathology, any systemic pathology or chronic pain, and any surgery in the last 6 months. Control group participants had to meet the same eligibility requirements as the dysmenorrhea group, except for not suffering from PD (with no more than a 3/10 on the Visual Analog Scale (VAS) during menstruation).

Sampling was conducted using call posters distributed to students and employees of the UEM. Interested participants reached out to our research team through the contact information provided on the posters and via referrals.

### 2.3. Sociodemographic Descriptive Data

During their first visit to the evaluation center, several baseline measurements were taken for the participating women. These included age, height, weight, Body Mass Index (BMI), smoking status, day of the menstrual cycle, and the VAS score for pain experienced during menstruation.

VAS for Pain: Participants rated their pain during menstruation on a 10-centimeter VAS line, with 0 indicating no pain and 10 representing the worst pain imaginable. After this, a ruler was used to measure the distance in centimeters from the zero mark to the patient’s pain mark. This provided a quantifiable measure of pain intensity.

After these initial assessments, participants were referred to the medical service to confirm a diagnosis of PD. A thorough medical evaluation was conducted to ensure that the dysmenorrhea was indeed primary and not secondary to any other underlying conditions. Once a diagnosis of PD was confirmed, the participants underwent further specific evaluations.

The subsequent evaluations included:

Abdominal Wall Assessment: Using ultrasound imaging to measure the thickness of the abdominal muscles (TrA, IO, EO, RA)

Anterior Pelvic Tilt Measurement: Conducted using the Palpation Meter (PALM) to determine the degree of pelvic anteversion.

Sagittal Plane Trunk Mobility: Assessed to evaluate the range of motion in trunk flexion and extension.

### 2.4. Ultrasound Assessment of Abdominal Wall Muscles

The ultrasound examination for image acquisition was performed by an experienced physiotherapist with more than five years of experience in musculoskeletal ultrasound. Participants were assigned a code before the start of the assessment, ensuring that the assessor was blinded to the group allocation (dysmenorrhea or control).

A state-of-the-art ultrasound system (LOGIQ S7 Expert, GE Healthcare, Chicago, IL, USA), equipped with a linear transducer ranging from 4 to 15 MHz (Broad-spectrum linear matrix array probe ML6-15 H40452LY, field of view of 50 mm), was utilized to conduct ultrasound imaging for the assessment of abdominal wall muscles. Scanning was performed on both the right and left sides to ensure a comprehensive evaluation, with three measurements taken at each site. The ultrasound probe was repositioned between each trial to enhance measurement reliability and accuracy [14,15].

For the assessment of abdominal muscles via ultrasound, standardized guidelines were meticulously followed [14,15]. To measure the resting thickness of the abdominal muscles, patients were positioned supine with slightly bent knees supported by a cushion to ensure comfort and consistency in positioning. During contraction measurements, patients were instructed to extend the opposite leg, performing the active straight leg raise (ASLR) test to engage the muscles appropriately.

The reference positions for transducer placement are outlined as follows [16,17].

For the transversus abdominis (TrA), internal oblique (IO), and external oblique (EO) muscles, The transducer was positioned at the midpoint between the iliac crest and the lower edge of the rib cage.

**Figure 1:**
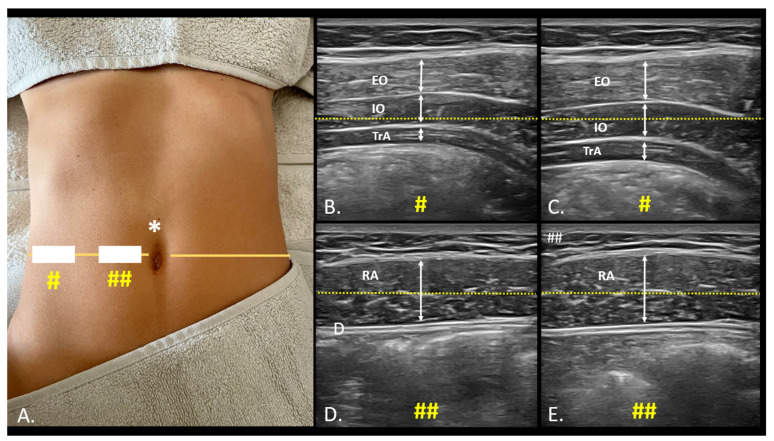
USI assessment and measures of anterolateral abdominal wall. Placement of the probe for the evaluation of the lateral muscles of the abdomen (#) and the rectus abdominis (##) using the umbilical line (*) as a reference (dotted lines) (**A**); Evaluation of changes in ultrasound thickness between rest (**B**,**D**) and muscle contraction (**C**,**E**) of the lateral muscles of the abdomen ((**B**,**C**), #) and the rectus abdominis ((**D**,**E**) ##). Abbreviations: EO, external oblique; IO, internal oblique; RA, rectus abdominis; TrA, transversus abdominis.

For the RA muscle: The transducer was positioned over the midpoint of the RA muscle, at the level of the umbilicus at the level of the umbilicus, ensuring a clear view of the muscle’s cross-section (Figure 1). 

For the TrA, IO, and EO muscles: The transducer was positioned halfway between the iliac crest and the lower edge of the rib cage, following the mid-axillary line. This placement allowed for an optimal view of the lateral abdominal muscles (Figure 1).

By employing these precise techniques and positions, we ensured that the ultrasound assessments provided accurate and reliable data for both the intrinsic foot muscles and the abdominal muscles.

### 2.5. Assessment of Anterior Pelvic Tilt

**Figure 2:**
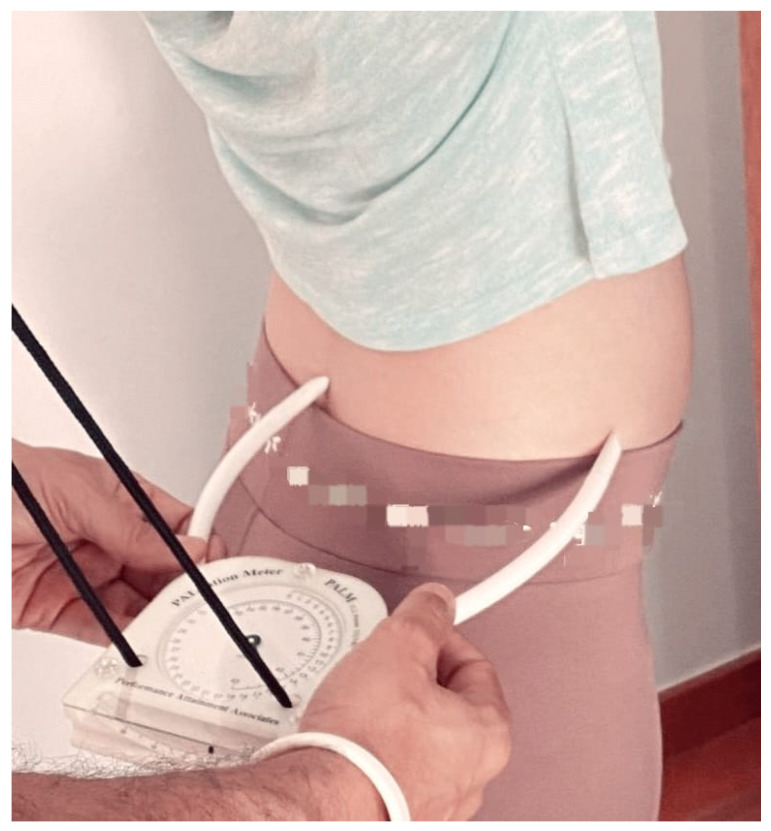
Assessment and measures of anterior. Pelvic tilt using the Palpation Meter.

The measurement of anterior pelvic tilt was conducted using the PALM, a reliable tool for assessing pelvic alignment. For this procedure, subjects were positioned in a standing posture with their feet shoulder-width apart to ensure a stable and natural stance. The posterior end of the PALM device was carefully placed on the Posterior Superior Iliac Spine (PSIS), while the anterior end was positioned over the Anterior Superior Iliac Spine (ASIS). This positioning allowed for an accurate measurement of pelvic angulation. The angle formed between these two anatomical landmarks indicates the degree of anterior pelvic tilt. This method is well-documented and referenced in previous studies [18], ensuring consistency and reliability in the measurement process (Figure 2).

### 2.6. Assessment of Trunk Mobility

Trunk flexion and extension range of motion (ROM) were meticulously assessed using the activForce2 accelerometer, a sophisticated device that accurately measures movements in degrees. This assessment aimed to provide precise and reliable data on the participants’ trunk mobility.

#### 2.6.1. Trunk Flexion

The evaluation of trunk flexion began with the participant standing upright, with feet shoulder-width apart, to ensure a stable base. Participants were then instructed to bend forward at the waist and attempt to touch their toes. The activForce2 accelerometer was strategically positioned at the level of the third lumbar vertebra (L3) to accurately capture the degree of forward bending. This position allowed for consistent and reliable measurement of trunk flexion. Each participant performed the movement three times, ensuring the measurements were reproducible and any variability was minimized.

#### 2.6.2. Trunk Extension

The evaluation of trunk extension also started with the participant standing upright, with feet shoulder-width apart and hands resting on their hips for balance. Participants were instructed to lean their trunk backward as far as possible, extending their spine. The activForce2 accelerometer was positioned at the level of the pubic symphysis to measure the degree of backward bending accurately. This setup ensured that the extension movements were captured precisely, providing reliable data on the participants’ trunk extension capacity. Similar to the flexion assessment, three measurements were recorded for each participant.

To ensure consistency and reduce the potential for muscle fatigue affecting the results, a resting period of 30 s was provided between consecutive measurements. This rest period allowed the participants’ muscles to recover briefly, promoting more accurate and consistent measurements across the three trials.

Using the activForce2 accelerometer and following a standardized procedure, we ensured that the assessment of trunk flexion and extension ROM was both precise and reliable, providing valuable insights into the participants’ trunk mobility.

### 2.7. Statistical Analysis

To determine the sample size, a pilot study with 20 participants was conducted, consisting of two groups using the RA thickness variable: 10 participants in each group. The variability of the RA thickness was used for the sample size calculation using the G*Power software. The following parameters were set: a confidence level of 95%, a statistical power of 0.80, an effect size of 0.87, and an α error of 0.05. Based on these parameters, the required sample size was determined to be 22 participants per group, resulting in a total sample size of 44 participants.

Data analysis was conducted using SPSS 29.0 software (IBM SPSS Statistics, IBM, Armonk, NY, USA). Quantitative variables were described using mean and standard deviation, while qualitative variables were reported using the number and percentage of participants. U Mann–Whitney test was carried out to analyze differences between the control and the dysmenorrhea groups. Cohen’s d was calculated to quantify the difference in muscle thickness, lumbopelvic alignment, and trunk mobility between women with PD and those without. Effect sizes were categorized as follows: 0.2 was considered small, 0.5 was medium, and 0.8 was large. Comparisons of qualitative data were tested using the chi-square test. The level of significance was set at ≤0.05.

## 3. Results

The study included a total of 44 women, with 22 participants in each group. Table 1 shows the characteristics of participants overall and distributed by study group. As can be observed, the two groups were homogeneous in the measured characteristics except for smoking, with statistically significant differences due to a higher number of smokers in the group with PD. As expected, statistically significant differences were also found between the groups regarding pain, with almost a 5-point difference in the VAS scale between groups.

Table 2 shows the thickness of the abdominal muscles in women with PD compared to those without dysmenorrhea. The RA muscle thickness at rest on the right side showed a significant difference (*p* = 0.026), with a moderate effect size (Cohen’s d = 0.75) and a mean difference of 0.13. The RA muscle thickness at rest on the left side also demonstrated a significant difference (*p* = 0.030), with a moderate effect size (Cohen’s d = 0.74) and a mean difference of 0.13. Additionally, during contraction on the left side, the RA muscle thickness showed a significant difference (*p* = 0.046), with a moderate effect size (Cohen’s d = 0.67) and a mean difference of 0.13.

The EO muscle thickness at rest on the left side showed a significant difference (*p* = 0.045), with a moderate effect size (Cohen’s d = 0.67) and a mean difference of 0.10. Moreover, during contraction on the left side, the EO muscle thickness had a highly significant difference (*p* = 0.003), with a large effect size (Cohen’s d = 0.97) and a mean difference of 0.18. These findings suggest substantial differences in the muscle thickness between the two groups, highlighting that PD is associated with selective weakening or thinning of certain superficial abdominal muscles, particularly the RA and EO. On the other hand, no significant differences were found for the IO and TrA muscles, either at rest or during contraction (*p* > 0.05).

Regarding trunk mobility and anterior pelvic tilt, the analysis revealed no significant differences between women with PD and those without trunk flexion (*p* = 0.897) and extension (*p* = 0.897). Similarly, no significant differences were observed in the measurements of anterior pelvic tilt between the two groups (*p* = 0.795) (Table 3).

## 4. Discussion

The results of the present study carried out to assess pelvic anteversion, trunk mobility (flexion-extension), and thickness of the abdominal muscles in women with PD compared to those without dysmenorrhea, indicated significant differences in the thickness of the RA and EO muscles. It was observed that women with PD have reduced thickness in these abdominal muscles with respect to those without dysmenorrhea.

### 4.1. Ultrasound Abdominal Measurements

These findings suggest that dysmenorrhea may be associated with selective weakening or thinning of certain superficial abdominal muscles, specifically the RA and EO. The decrease in muscle thickness may be related to chronic pain and reduced physical activity in women with dysmenorrhea, potentially leading to muscle atrophy in these specific areas. This hypothesis aligns with previous studies that have reported structural alterations in muscles in the presence of chronic pain, such as muscle atrophy, morphological changes, or fat infiltration [19,20].

A possible explanation for the changes in superficial musculature is that chronic pain, such as that experienced in dysmenorrhea, can lead to structural modifications and changes in muscle activity as a compensatory mechanism, especially in muscles that actively participate in posture and movement [21,22]. Changes in estrogen and progesterone levels can influence fluid retention and muscle elasticity, mainly affecting muscles exposed to mechanical loads. This link between hormonal changes and muscle properties is supported by previous research indicating that hormones such as progesterone can modify muscle response to mechanical stretch, thus affecting muscle function and structure. In this sense, the study by Mitchell et al. [23] examined how mechanical stretch and hormone regulation can affect smooth muscle cells, highlighting the complex interactions between hormone levels and muscle properties.

On the other hand, the absence of significant changes in the thickness of the deeper muscles, such as TrA and OI, suggests that PD may not affect the deep abdominal musculature in the same way. This could be due to the different roles these muscles play in trunk stability and posture compared to the superficial muscles that are more involved in abdominal movement and strength. Our results are consistent with a previously published study that also found no differences in abdominal wall thickness in women with dysmenorrhea versus a control group; however, in those cases, they observed that there was no difference in the thickness of the TrA and IO muscles, but also no difference in the RA and EO between groups [24].

Additionally, the absence of significant changes in the deep musculature is consistent with the observations of Stuge et al. [25], who examined the function of abdominal and pelvic floor muscles (PFM) in postpartum women with long-term pelvic girdle pain compared to the control group. In their study, they also did not find significant differences in the thickness of the deep abdominal muscles (TrAb and IO) or PFM strength. These findings suggest that the ability to voluntarily contract the deep abdominal muscles and the strength of the PFMs are not associated with persistent pelvic pain.

It is important to note that no changes were observed in the alignment of pelvic anteversion or the mobility of trunk flexion and extension. The TrA and IO muscles, which showed no significant differences in thickness, play a crucial role in the stability of the trunk and pelvis. Their integrity may be sufficient to maintain pelvic alignment and trunk mobility despite the differences observed in superficial muscles like the RA and EO.

It is possible that women with dysmenorrhea develop compensatory mechanisms that preserve the structure, posture, and mobility of the trunk, even when some superficial abdominal muscles are weakened. These mechanisms may include the synergistic use of other muscles or postural adjustments that do not evidently affect pelvic alignment or trunk mobility.

### 4.2. Lumbopelvic Alignment and Trunk Mobility

In the present study, no significant differences were found in lumbopelvic alignment between women with PD and asymptomatic women. This result suggests that pelvic alignment alone may not be a determining factor in PD. However, there is no consensus in the scientific literature regarding the relationship between pelvic alignment and PD. Kim et al. [26] used the Formetic 4D device to examine lumbopelvic position and found no significant differences in the pelvic tilt variable between the dysmenorrhea group and the control group; however, they found that scoliosis and lumbar lordosis increased in the PD group. Similarly, the study by Karakus et al. [11] found no differences in thoracic, lumbar, and pelvic angles in the sagittal and frontal planes between the dysmenorrhea group and the asymptomatic group, which coincides with our results. In contrast, other studies observed structural changes such as pelvic torsion [27], which detected greater pelvic torsion in women with dysmenorrhea compared to healthy women, but they did not observe changes in anteversion.

A recent study investigated the relationship between dysmenorrhea and sagittal spinopelvic alignment. The results showed significant differences in several spinopelvic parameters between groups: pelvic incidence, sacral slope, lumbar lordosis, and thoracic kyphosis. Additionally, a significant negative correlation was found between pain level and sacral slope in the dysmenorrhea group [27]. Differences in findings between studies may be due to measurement methods, sample characteristics, and other contextual factors. 

In conclusion, although our results do not support a significant relationship between lumbopelvic alignment and PD, more research is needed to fully understand the possible underlying mechanisms and clinical implications of lumbopelvic alignment in menstrual health. 

Furthermore, no significant differences in trunk mobility were detected when assessed in the sagittal plane between the two groups. One possible explanation for this finding is that trunk mobility, measured in terms of flexion and extension, may not be directly affected by the presence of PD. It is possible that although PD predominantly affects the lumbar and pelvic regions and induces localized pain, it does not significantly alter overall trunk mobility. Another consideration is that compensatory mechanisms in other muscle groups may help maintain trunk mobility, even in the presence of pelvic pain. Future research could explore whether movements in other planes or a combination of movements could reveal more subtle differences or uncover the creation of potential compensatory patterns in women with PD.

Nonetheless, this study provides new insights into how PD affects different muscle groups, suggesting that superficial muscles are more involved in the pain response. Therapeutic interventions could include specific exercises to strengthen and relax superficial muscles, such as the RA and EO muscles, potentially reducing pain and improving posture. 

These results can help develop more effective and specific treatment strategies for women suffering from dysmenorrhea. Future studies should consider including hormonal and menstrual cycle factors in their analysis.

### 4.3. Limitations

The sample used in the study was specific and did not include factors of ethnic and socioeconomic diversity that possibly affected the generalization of the results. The implication of this limitation is the fact that the findings may not be applied to a wider and more varied population, therefore indicating that future study designs should include a wider spectrum of demographic diversity. Additionally, participants were recruited from a single university, which may not represent a broader, more diverse population. 

Controlling external factors, the majority of which, such as physical activity and postural habits, likely have a significant influence on muscle structure, has not been considered. As these latter factors can significantly affect the results of the study, not looking at them does not provide a full understanding of the relationship of dysmenorrhea with the changes in the musculoskeletal system.

The possible psychosocial factors that might affect responsiveness to pain and musculoskeletal health, such as stress, anxiety, or depression, were not considered in the study. The evaluation of such factors may enhance the understanding of the contributors of dysmenorrhea and its effects on the musculoskeletal system. 

Importantly, the low number of female smokers in the present study did not allow us to attempt to analyze if there was a correlation between pain and smoking, as other studies have suggested [28,29]. Smoking, which is significantly higher in the PD group, may introduce confounding variables that affect the parameters assessed. Smoking influences vascular health and inflammation, potentially impacting the body. Recognizing these differences helps us interpret our findings accurately.

Although validated, these methods may not capture all relevant anatomical and postural differences. Perhaps methods different than those used in the current measurement or more comprehensive assessment tools might provide a fuller picture of the musculoskeletal alterations related to PD. Furthermore, other potential factors related to PD, such as muscle strength, flexibility, and hormonal influences, were not explored.

Pain was assessed as self-reported, and this can introduce subjective biases in dysmenorrhea assessment. Levels of self-reported pain can vary considerably and may not always reflect the objective severity of the condition. Furthermore, they did not control for hormonal levels or the respective phases of the menstrual cycle. As hormonal changes affect muscle efficiency and pain sensitivity, further research needs to conduct hormonal tests to have more clarity on this role. Although the present study was conducted with a relatively modest sample size of n = 44 to achieve a target level of statistical power, this sample size may not be sufficient to detect smaller but potentially clinically significant differences between groups. A larger sample size in future studies can notably boost the strength and robustness of the findings. 

The research was cross-sectional and permits the observation of the relationship at one point in time; it does not show how the relationship between dysmenorrhea and musculoskeletal parameters changes over a period. A longitudinal study design would be useful to show how these observations change over time and establish causal relationships.

Finally, the study did not examine the functional impact of the observed differences in muscle thickness on daily activities and quality of life. Future research should address these limitations to provide a more comprehensive understanding of the musculoskeletal aspects of PD.

## 5. Conclusions

The results revealed that significant changes (*p* < 0.05) were only observed in the musculature of the RA and the EO muscles with lower values in the woman with PD versus the control group, while no statistically significant differences (*p* > 0.05) were found in the musculature of the TrA or the IO. 

Additionally, no significant differences (*p* > 0.05) were detected in lumbopelvic alignment or trunk mobility in flexion and extension between the two groups.

Our findings suggest that PD is not associated with alterations in lumbopelvic alignment or trunk mobility but primarily affects the superficial abdominal musculature. These results can guide future therapeutic strategies towards strengthening and relaxing the superficial muscles, such as the RA and EO, for managing pain in women with PD.

## Figures and Tables

**Table 1:** Characteristics of study subjects overall and by study group.

Variable	All Subjects (n = 44)	Control Group (n = 22)	Dysmenorrhea Group (n = 22)	*p*
Age	26.16 ± 6.97	25.14 ± 7.41	27.18 ± 6.51	0.153
Height	164.91 ± 6.46	163.82 ± 6.86	166.00 ± 5.98	0.188
Weight	61.24 ± 9.88	61.16 ± 10.89	61.32 ± 9.02	0.778
BMI	22.44 ± 2.97	22.70 ± 3.31	22.18 ± 2.64	0.725
Smoking, n (%)	9 (20.45)	1 (4.55)	8 (36.36)	**0.021**
Dominant side—Right, n (%)	40 (90.90)	20 (90.90)	20 (90.90)	1.00
Pain (VAS)	5.68 ± 2.81	3.23 ± 1.38	8.14 ± 1.28	**<0.001**
Day of the menstrual cycle	15.30 ± 7.40	14.05 ± 7.38	16.55 ± 7.39	0.254

BMI: Body mass index (kg/m^2^). Data are shown as mean ± SD except where indicated.

**Table 2:** Thickness (cm) of the abdominal muscles in women with primary dysmenorrhea compared to those without dysmenorrhea.

Variable	Control Group (n = 22)	Dysmenorrhea Group (n = 22)	*p*	Mean Difference (95% CI)	Effect Size (Cohen’s d)
**Right RA**					
At rest	1.08 ± 0.19	0.95 ± 0.14	**0.026**	0.13 (0.02, 0.24)	0.75
In contraction	1.13 ± 0.18	0.99 ± 0.18	0.118	0.14 (0.03, 0.25)	0.78
**Left RA**					
At rest	1.07 ± 0.19	0.94 ± 0.14	**0.03**	0.13 (0.02, 0.24)	0.74
In contraction	1.11 ± 0.20	0.98 ± 0.17	**0.046**	0.13 (0.02, 0.24)	0.67
**Right EO**					
At rest	0.73 ± 0.20	0.65 ± 0.14	0.24	0.08 (0.02, 0.18)	0.45
In contraction	0.83 ± 0.16	0.75 ± 0.18	0.084	0.08 (0.03, 0.17)	0.46
**Left EO**					
At rest	0.72 ± 0.18	0.62 ± 0.11	**0.045**	0.10 (0.01, 0.19)	0.67
In contraction	0.87 ± 0.21	0.69 ± 0.14	**0.003**	0.18 (0.08, 0.28)	0.97
**Right IO**					
At rest	0.78 ± 0.16	0.73 ± 0.16	0.431	0.05 (0.01, 0.09)	0.31
In contraction	0.90 ± 0.20	0.82 ± 0.25	0.127	0.08 (0.02, 0.18)	0.34
**Left IO**					
At rest	0.76 ± 0.17	0.68 ± 0.13	0.18	0.08 (0.02, 0.18)	0.49
In contraction	0.86 ± 0.21	0.77 ± 0.14	0.13	0.09 (0.03, 0.21)	0.45
**Right TrA**					
At rest	0.33 ± 0.12	0.32 ± 0.07	0.833	0.01 (−0.03, 0.05)	0.1
In contraction	0.43 ± 0.16	0.40 ± 0.12	0.614	0.03 (−0.01, 0.07)	0.2
**Left TrA**					
At rest	0.32 ± 0.09	0.31 ± 0.09	0.417	0.01 (−0.03, 0.05)	0.11
In contraction	0.44 ± 0.13	0.40 ± 0.12	0.28	0.04 (−0.01, 0.09)	0.32

RA: rectus abdominis; EO: external oblique; IO: internal oblique; TrA: transversus abdominis. Data are shown as mean ± SD.

**Table 3:** Pelvic anteversion and sagittal plane mobility (trunk flexion-extension) by study group.

Variable	Control Group (n = 22)	Dysmenorrhea Group (n = 22)	*p*	Mean Difference (95% CI)	Effect Size (Cohen’s d)
ROM Flex (°)	60.39 ± 23.35	60.01 ± 15.36	0.897	0.38 (−10.62, 11.38)	0.02
ROM Ext (°)	20.60 ± 10.80	19.98 ± 7.27	0.897	0.62 (−4.39, 5.63)	0.06
Anterior pelvic tilt (°)	7.69 ± 2.98	7.95 ± 2.82	0.795	−0.26 (−1.79, 1.27)	−0.09

° = degrees. ROM: Range of motion. Data are shown as mean ± SD.

## Data Availability

The datasets supporting the findings of this study can be requested from the first author.
